# Effect of pre-treatment systemic antibiotics on post-operative pain following single-visit root canal therapy in vital mandibular first premolars: A randomized controlled trial

**DOI:** 10.6026/973206300222910

**Published:** 2026-05-31

**Authors:** Shefali Talekar, Shivam Arora, Amit Patil, Sumita Bhagwat, Ankita Khose, Sanpreet Singh Sachdev

**Affiliations:** 1 Department of Conservative Dentistry and Endodontics, Bharati Vidyapeeth (Deemed to be University) Dental College and Hospital, Belapur CBD, Navi Mumbai, Maharashtra, India; 2 Department of Radiodiagnosis, MGM Medical College and Hospital, Vashi, Navi Mumbai, Maharashtra, India; 3 Department of Conservative Dentistry and Endodontics, DY Patil University School ofDentistry, Nerul, Navi Mumbai, Maharashtra, India; 4 Department of Oral Pathology and Microbiology, Bharati Vidyapeeth (Deemed to be University) Dental College and Hospital, Belapur CBD, Navi Mumbai, Maharashtra, India

**Keywords:** Amoxicillin, endodontic pain, single-visit endodontics, analgesic effect, antibiotic prophylaxis, randomized controlled trial

## Abstract

Most post-endodontic pain can be effectively managed pharmacologically without antibiotic use. However, some clinicians continue to prescribe antibiotics prophylactically, driven by concern over flare-ups or patient expectations. Therefore, it is of interest to evaluate the effect of pre-treatment systemic antibiotic prophylaxis on post-operative pain following single-visit root canal therapy. A randomized controlled trial was conducted involving 60 systemically healthy patients requiring root canal treatment in vital mandibular first premolars. Participants were divided into two groups: Group A received a single pre-operative dose of 500 mg amoxicillin one hour prior to treatment, while Group B received no antibiotic. Subjects who received antibiotics reported significantly lower pain scores during the initial 24 hours compared to the non-antibiotic group (p < 0.05). Thus, pre-operative systemic antibiotics may provide early post-operative pain relief in vital teeth, but antibiotic use should be judicious and reserved for specific clinical indications.

## Background:

Post-operative pain following root canal therapy remains a critical determinant of patient satisfaction and trust in dental care [1]. Discomfort after endodontic treatment can negatively affect the patient's perception of the dentist's competence and deter them from seeking future care. Pain-related fear is a significant barrier to routine dental attendance, with over 50% of adults citing it as the primary reason for avoiding dental visits [2]. Post-treatment pain also has psychological ramifications, including anxiety and reduced treatment compliance [3]. The origin of post-operative endodontic pain is multifactorial, typically arising from chemical, microbial and mechanical irritation of periapical tissues. Apical extrusion of debris, bacterial by-products or over-instrumentation is common contributors [4, 5]. Despite advances in endodontic materials and techniques, pain following treatment persists in up to 58% of patients [4]. Management strategies include the use of anti-inflammatory agents, analgesics and occasionally, antibiotics [6]. Among these, the pre-emptive use of antibiotics is particularly debated. While antibiotics are indispensable for managing systemic odontogenic infections, their routine use in vital pulp cases lacks strong evidence. Inappropriate prescription has been linked to antimicrobial resistance, allergic reactions and gastrointestinal distress [7, 8]. There is an emphasis on the shift toward antibiotic stewardship and evidence-based practice [9]. In endodontics, pain management should prioritize diagnosis, definitive treatment and drugs starting with NSAIDs or acetaminophen [10]. Opioids or combination therapy should be reserved only for severe cases [6, 11]. Most post-endodontic pain can be effectively managed pharmacologically without antibiotic use. However, some clinicians continue to prescribe antibiotics prophylactically, driven by concern over flare-ups or patient expectations. Studies assessing the efficacy of such practices have yielded inconsistent results, with some demonstrating mild benefit and others reporting no significant difference in pain levels [12,13]. Therefore, it is of interest to assess post-operative pain levels in vital mandibular first premolars treated with single-visit root canal therapy.

## Materials and Methods:

The present randomized controlled trial was conducted across a period of six months from July 2024 to December 2024. All procedures were performed in compliance with the ethical principles outlined in the Declaration of Helsinki. The clinical protocol was approved by the Institutional Research and Ethical Board (Ref ID: IREB/2021/ENDO/02, Dated 06/04/2021). Each patient was provided with a detailed explanation of the study objectives and methodology and written informed consent was obtained.

## Sample selection and inclusion criteria:

Participants were recruited from patients reporting with a clinical diagnosis of irreversible pulpitis in mandibular first premolars and were screened for eligibility. Systemically healthy patients with carious mandibular first premolars with a Visual Analog Scale (VAS) pain score of 7 or above were included [14]. Only those who responded positively to electric pulp testing and cold testing and demonstrated straight root canals were included. Patients were excluded if the affected teeth exhibited large restorations, existing prostheses, or previous endodontic treatment. Also, those having periodontal disease, pulp necrosis, or periapical pathology exceeding 2 mm were excluded. Teeth with curved or calcified canals were also excluded to maintain procedural consistency.

## Vitality testing:

To confirm pulpal vitality, each patient underwent diagnostic evaluation using an electric pulp tester (Denjoy DY3-10; Denjoy Dental Co., Ltd., Changsha, China). A cold stimulation test using an icy spray refrigerant (Detax GmbH & Co. KG, Ettlingen, Germany) was also performed to confirm pulp vitality. Positive responses to both tests, followed by bleeding on accessing the pulp chamber, were taken as confirmation of vital pulp tissue. Cases with no bleeding or negative responses were excluded.

## Randomization and group allocation:

The 60 patients were randomly divided into two groups of 30 each by simple randomization using computer-generated codes. Group A (antibiotic group) received a course of 500 mg amoxicillin plus 125 mg clavulanic acid tablets (Augmentin, Alkem Laboratories Ltd., Mumbai, India). The tablets were taken twice daily for three days prior to the scheduled root canal treatment and on the day of treatment. Group B (control group) did not receive any antibiotics prior to treatment. Both groups were administered a pre-operative dose of ibuprofen 400 mg (Ibugesic-200, Cipla Ltd., Mumbai, India) one hour before the procedure.

## Clinical procedure:

On the day of the treatment, local anaesthesia was administered using 2% lignocaine with 1:80,000 adrenalines (Indoco Remedies Ltd., Mumbai, India). Teeth were isolated using a rubber dam kit (Hygenic; Coltene Whaledent, Altstätten, Switzerland). The access cavity preparation was performed using a contra-angled high-speed airotor handpiece (Allure; Dental Avenue, Mumbai, India). A round and flat-end tapered diamond bur was used (Mani Inc., Tochigi, Japan). Working length determination was carried out using an apex locator (Root ZX; J. Morita Corp., Kyoto, Japan) and confirmed with a digital intraoral radiograph. Root canal instrumentation was done using the crown-down technique with rotary files (E3 Azure, sizes 20/04 and 25/04) and Gates Glidden drills (Mani Inc., Tochigi, Japan). Stainless steel K-files (sizes 10-40; Mani Inc., Japan) were used for apical patency and initial negotiation. RC Prep (Prime Dental Products Pvt. Ltd., Thane, India) was used as a canal lubricant. Canal irrigation was performed using 2.5% sodium hypochlorite solution (Prime Dental Products Pvt. Ltd., Thane, India) and normal saline. The solution was delivered using disposable 2 mL syringes and a 30-gauge side-vented irrigation needle (United Dental Group, Zhengzhou, China). An endoblock (Dentsply Sirona, York, USA) was used to standardize file length. Following complete biomechanical preparation, the canals were dried with sterile absorbent paper points. Obturation was performed using gutta-percha cones with Sealapex™ root canal sealer (Kerr Corporation orange, CA, USA) via the lateral condensation technique. Temporary restoration containing a zinc oxide-based temporary filling material (Tempfil-G; Shivam Dental Products, Mumbai, India). A confirmatory radiograph was obtained to confirm the quality of final obturation.

## Pain assessment protocol: 

Post-operative pain was assessed using the VAS, where patients recorded their pain scores at seven time intervals. These were at 4 hours, 8 hours, 12 hours, 24 hours, 48 hours, 72 hours, and 7 days after completion of the root canal treatment. Pain intensity was categorized as follows: No pain (score 0), mild (1-4), moderate (5-7), and severe (8-10). Patients were provided with a printed questionnaire on the day of treatment and instructed to return it at their 7-day recall visit.

## Post-treatment protocol and follow-up:

All patients were prescribed a rescue analgesic (ibuprofen 400 mg) to be taken only if necessary. At the 7-day follow-up visit, completed pain questionnaires were collected and any complications or additional analgesic intake were recorded. Fifteen days after obturation, patients received definitive post-endodontic restorations under standard protocols.

## Statistical analysis:

The collected data were compiled and analyzed using SPSS software version 25.0 (IBM Corp., Armonk, NY, USA). Descriptive statistics, including frequencies, percentages, means and standard deviations, were calculated for demographic variables and pain scores. Intergroup comparisons of pain scores at each time interval were performed using the Mann-Whitney U test due to non-parametric distribution. The incidence of pain (presence versus absence) between groups was analyzed using the Chi-square test and Fisher's exact test was applied. A p-value < 0.05 was considered statistically significant for all tests.

## Results:

**Figure 1:**
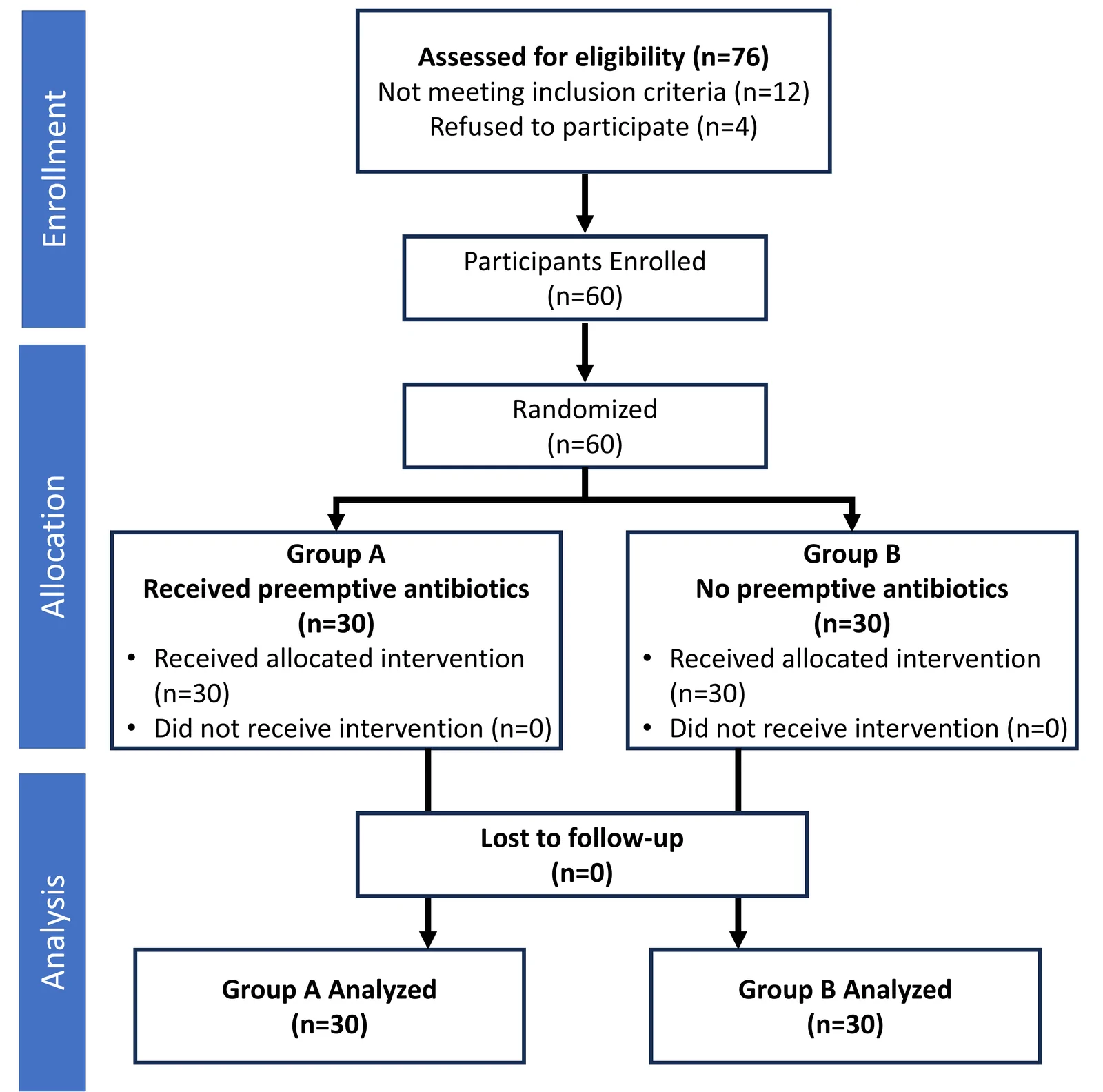
CONSORT Flow diagram indicating the flow of the participants form recruitment to analysis

The present study included a total of 60 adult patients requiring endodontic treatment in vital mandibular first premolars, with an age range of 18 to 60 years. The CONSORT Flow diagram indicating the study process is depicted in Figure 1. The mean age of the participants was 37.52 ± 11.38 years. Among them, 27 were males and 33 were females. The sample was distributed across four age categories: 20 participants were aged 18-30 years, 12 were between 31-40 years, 11 were between 41-50 years and 17 were aged 51-60 years. The demographic characteristics were statistically comparable between Group A (with antibiotics) and Group B (without antibiotics). There was no significant difference in the distribution of participants by age group or gender between the two study groups (p > 0.05); indicating that age and gender did not act as confounding variables in the assessment of post-operative pain outcomes. Pain incidence and intensity were recorded using the VAS at baseline and at multiple post-operative time intervals up to seven days. The total number of patients reporting post-operative pain (VAS > 0) was consistently lower in Group A (with antibiotics) compared to Group B (without antibiotics), particularly within the first 24 hours (Table 1). Statistically significant differences (p<0.05) were observed at 4 hours (p = 0.0199) and 8 hours (p = 0.0159), with Group A showing fewer pain reports. The differences at 12 hours (p = 0.0608) and 24 hours (p = 0.0651) approached but did not reach statistical significance. No significant difference was found at 48 hours (p = 1.0000) and no patients reported pain on the 3rd and 7th days in either group. The pre-operative pain scores were comparable between the two groups, confirming a uniform baseline. Post-operatively, during the first 24 hours, Group A (with antibiotics) reported consistently lower pain scores than Group B (without antibiotics). The mean VAS score at 4 hours was 0.40 ± 0.67 in Group A and 1.67 ± 1.90 in Group B; This difference was statistically significant (p = 0.0013). Similar trends were observed at 8 hours (p = 0.0013), 12 hours (p = 0.0022) and 24 hours (p = 0.0134), favoring the antibiotic group. However, by 48 hours, pain scores had decreased in both groups and the difference was no longer statistically significant (p = 0.7295). All patients in both groups reported complete pain resolution at 3 and 7 days. The comparison of post-operative pain levels between the two groups at various time intervals is presented in Table 2. The results indicated a statistically significant reduction in post-operative pain in the antibiotic group during the first 24 hours following treatment. Beyond this period, the difference in pain levels between the groups diminished and was not significant by 48 hours or later. Pain severity was categorized as no pain (VAS = 0), mild pain (VAS 1-4), moderate pain (VAS 5-7) and severe pain (VAS 8-10). A higher proportion of patients in Group A (with antibiotics) reported no pain or only mild discomfort during the early post-operative period, whereas Group B showed a greater number of patients with moderate and severe pain, particularly within the first 12 hours. At 4 hours, 70% of patients in Group A experienced no pain compared to 36.7% in Group B, a difference that was statistically significant (p<0.05). Similar significant differences were noted at 8 hours (p = 0.0159). However, differences in severity distribution at 12 hours (p = 0.0608), 24 hours (p = 0.0651) and 48 hours (p = 1.0000) were not statistically significant (p>0.05). By 3 days, all patients in both groups were pain-free. The comparative distribution of participants based on categorical pain levels is summarized in Table 3. These findings confirm that antibiotic prophylaxis was associated with significantly (p<0.05) reduced severity of post-operative pain in the first eight hours following root canal treatment. However, beyond this period, pain levels declined in both groups and the difference in pain severity became statistically insignificant (p>0.05).

## Discussion:

The present study investigated the influence of pre-treatment systemic antibiotic prophylaxis on post-operative pain following single- visit root canal therapy. The findings revealed that subjects who received antibiotics reported significantly lower pain intensity at 4, 8, 12 and 24 hours post-operatively compared to those who did not. These results support the hypothesis that systemic antibiotics may offer short-term benefits in modulating the early inflammatory response following endodontic instrumentation in vital teeth [15]. The outcome partially aligns with previous reports demonstrating a transient reduction in post-operative discomfort with pre-operative antibiotic coverage. For instance, a significant decrease in pain scores was observed within the first 24 hours in a previous study in which pre-endodontic amoxicillin was administered [16]. Literature has reported early pain relief benefits with antibiotics, although long-term differences were sparse [17]. Our study supports these early-phase findings but further confirms that this benefit is not sustained beyond 48 hours. No significant difference in pain was noted after this period and pain incidence dropped to zero by the third and seventh post-operative days in both groups. In contrast, several other studies have challenged the need for prophylactic antibiotics in endodontics, particularly in vital cases [18, 19]. Pathak *et al.*(2021) reported no statistically significant pain reduction following antibiotic administration. They emphasized the risks associated with unnecessary antibiotic use, including microbial resistance and adverse reactions [20]. This divergence in the literature highlights the need to contextualize antibiotic prescription within patient-specific risk factors, systemic health and treatment complexity. In the present study, the observed reduction in pain could be attributed to the systemic anti-inflammatory effects of the antibiotics. Additional factors include lowered bacterial translocation during instrumentation and pre-emptive suppression of microbial proliferation at the apical area. The specific pharmacological mechanism of amoxicillin in this context may further support the clinical findings. Amoxicillin acts by inhibiting bacterial cell wall synthesis and is rapidly absorbed, achieving therapeutic concentrations in plasma and gingival crevicular fluid [21]. This may help curtail bacterial presence and suppress the downstream release of nociceptive inflammatory mediators, such as prostaglandins and interleukins. Its early systemic availability could explain the reduced pain perception during the acute post-operative phase in the antibiotic group. However, while the findings provide evidence for a transient analgesic benefit of antibiotics, it must be interpreted cautiously. A key limitation of the study is its relatively small sample size and restriction to vital single-rooted teeth, which may not reflect outcomes in multicoated teeth or necrotic pulps. Furthermore, all participants were systemically healthy, potentially limiting the applicability to medically compromised patients. The use of subjective VAS scores for pain assessment, though widely validated, can be influenced by patient psychology, anxiety and prior experiences. Also, microbiological samples or inflammatory markers were not evaluated to objectively correlate pain with microbial or immunological parameters. An additional consideration pertains to the ethical and public health implications of antibiotic stewardship. While short-term symptomatic relief is desirable, it must be weighed against the long-term risk of fostering antibiotic resistance. Clinicians are urged to remain judicious in their prescribing practices and to prioritize non-antibiotic strategies when appropriate [11]. The present study, therefore, highlights the delicate balance between optimizing patient comfort and upholding responsible antimicrobial usage. Future research should involve larger multicenter trials with diverse tooth types and systemic profiles to validate the generalizability of these findings. Evaluating the effect of different antibiotic regimens, doses and timing would help provide a clearer understanding of the biological rationale behind pain modulation. Studies investigating localized drug delivery systems could serve as alternatives that minimize systemic risks while optimizing patient comfort.

## Conclusion:

We show that pre-treatment with systemic antibiotics may provide a transient advantage in alleviating early post-operative discomfort following endodontic therapy in vital teeth. However, the clinical relevance of this benefit must be carefully balanced against the principles of antibiotic stewardship. Thus, clinicians are encouraged to critically evaluate the need for systemic antibiotics in routine endodontics and to explore alternative, evidence-based strategies for effective pain management.

## Figures and Tables

**Table 1:** Number of patients reporting post-operative pain (VAS > 0) at different time points with statistical comparison between groups

**Time Interval**	**Group A (n)**	**Group B (n)**	**Test Statistic**	**p-value**
4 Hours	9	19	5.42	0.0199*
8 Hours	14	24	5.81	0.0159*
12 Hours	15	23	3.52	0.0608
24 Hours	8	16	3.4	0.0651
48 Hours	4	5	N/A	1
3 Days	0	0	N/A	N/A
7 Days	0	0	N/A	N/A
Chi-square test
for 4 Hours to 24 Hours;
Fischer's Exact test
for 48 Hours;
N/A = not applicable;
*Statistically significant
at p < 0.05

**Table 2:** Comparison of post-operative pain scores between groups at different time intervals

**Time Interval**	**Group A **	**Group B (Mean ± SD)**	**U value**	**p-value**
4 Hours	0.40 ± 0.67	1.67 ± 1.90	250.5	0.0013*
8 Hours	0.87 ± 1.17	2.03 ± 1.43	239.5	0.0013*
12 Hours	0.87 ± 1.07	2.20 ± 1.77	249.5	0.0022*
24 Hours	0.40 ± 0.72	1.30 ± 1.58	302	0.0134*
48 Hours	0.17 ± 0.46	0.23 ± 0.63	435	0.7295
Mann-Whitney U Test;
*Statistically significant
at p < 0.05

**Table 3:** Pain severity distribution and intergroup comparison

**Time Point**	**Group**	**No Pain (%)**	**Mild Pain (%)**	**Moderate Pain (%)**	**Severe Pain (%)**	**χ** ^2^	**p-value**
4 hours	A	70	30	0	0	5.42	0.0199*
	B	36.7	56.7	3.3	3.3		
8 hours	A	53.3	46.7	0	0	5.81	0.0159*
	B	20	76.7	3.3	0		
12 hours	A	50	50	0	0	3.52	0.0608
	B	23.3	63.3	13.4	0		
24 hours	A	73.3	26.7	0	0	3.4	0.0651
	B	46.7	50	3.3	0		
48 hours	A	86.7	13.3	0	0	0	1
	B	83.3	16.7	0	0		
